# Accuracy of a Text Intervention to Minimize the Burden of Cancer Care Among Patients Treated With Immune Checkpoint Inhibitors

**DOI:** 10.1001/jamanetworkopen.2022.28452

**Published:** 2022-08-29

**Authors:** Erin M. Bange, Kerry Coughlin, Wenrui Li, Elizabeth Moriarty, Timothy J. Brown, Lawrence N. Shulman, Ronac Mamtani

**Affiliations:** 1 Abramson Cancer Center, University of Pennsylvania, Philadelphia; 2 Penn Center for Cancer Care Innovation, University of Pennsylvania, Philadelphia; 3 Department of Biostatistics, Epidemiology and Informatics, University of Pennsylvania, Philadelphia

## Abstract

This cross-sectional study examines whether patients with cancer without symptoms of immune checkpoint inhibitor toxic effects can be accurately identified using a text message–based triage instrument and safely proceed to treatment.

## Introduction

Patients with cancer spend substantial time receiving cancer care^1^; thus, innovative strategies to decrease the time burden of cancer therapy are needed. The current care model consists largely of in-person visits to assess clinical status and treatment-related toxic effects. However many patients treated with immune checkpoint inhibitors (ICIs) do not experience toxic effects.^2^ We hypothesized that these patients without symptoms of ICI toxic effects could be accurately identified using a text message–based triage instrument and safely proceed to treatment, lessening the need for an in-person visit.

## Methods

This single-center, cross-sectional study evaluated the performance characteristics of a text message–based triage instrument to identify patient-reported ICI toxic effects, against the standard in-person clinician assessment documented in the electronic medical record. Patients were consecutively screened, and those who spoke English, were receiving single-agent ICI for a solid tumor, and had access to text messaging were approached for oral consent. Performance characteristics and number needed to text for 1 additional patient to avoid an office visit, were calculated with 95% CIs (Figure). The instrument contained 16 questions adapted from the National Cancer Institute’s Professional Common Terminology Criteria for Adverse Events (eAppendix in the Supplement) and was administered once at a single point in time during their treatment course via secure text message 96 hours before the patient’s scheduled infusion. A positive response to any question (eg, score >0) was used to indicate toxic effects (eAppendix in the Supplement). Clinic notes were reviewed by 2 trained personnel (E.M. and T.J.B.) for the presence or absence of any grade of ICI toxic effect. Patient perspectives were quantified (eAppendix in the Supplement). This study was approved by the University of Pennsylvania international review board and followed the STROBE reporting guideline.

**Figure.**
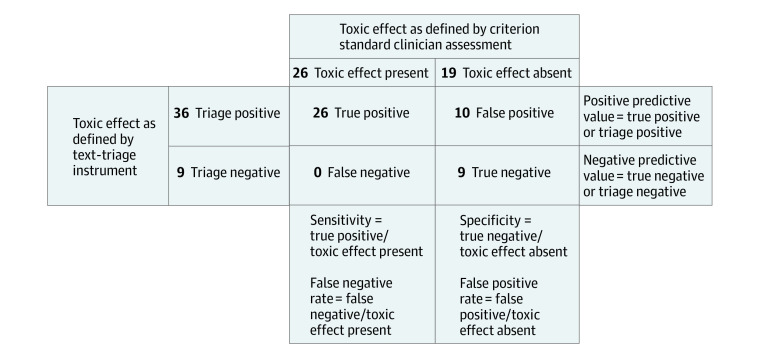
Calculation of Performance Characteristics of Instrument to Identify Patient-Reported Immunotherapy Toxic Effects

## Results

Between October 1 and November 25, 2021, 70 patients were approached for consent. Of these, 50 enrolled and 45 patients completed the instrument. The median (IQR) age was 68 (60-72) years, 31 (62%) were male, and 44 (88%) were White. The median (IQR) ICI cycle number was 12.5 (4.0-23.0); most patients received either pembrolizumab (27 patients [54%]) or nivolumab (17 patients [34%]) for palliative management (37 patients [74%]) of genitourinary (15 patients [30%]), lung (13 patients [26%]), or skin (11 patients [22%]) cancers. Patients who completed the instrument were more likely to be younger (median [IQR] age, 67 [60-71] vs 76 [73-76] years) and male (30 patients [66%] vs 15 patients [33%]) than those who did not.

The prevalence of any documented ICI toxic effects in the electronic medical record was 57.8%. The instrument had 100% sensitivity (95% CI, 87%-100%) corresponding to a 0% false-negative rate, 47% specificity, and a negative predictive value of 100% (95% CI, 66%-100%). The number needed to text was determined to be 5 patients. Other accuracy parameters are presented in the Table. Visual impairment and limited access to a smartphone were common reported barriers to completion.

**Table.** Performance Characteristics of Instrument to Identify Patient-Reported Immunotherapy Toxic Effects

Performance metric	Estimate (95% CI)^a^
Sensitivity	1.000 (0.868-1.000)
Specificity	0.474 (0.244-0.711)
PPV	0.722 (0.548-0.858)
NPV	1.000 (0.664-1.000)
NNT^b^	5.000 (2.891-10.443)

Abbreviations: NNT, number needed to text; NPV, negative predictive value; PPV, positive predictive value.

^a^ The 95% CIs for sensitivity, specificity, PPV, and NPV, and risk difference were calculated by the Clopper-Pearson method. The 95% CI for NNT was obtained by inverting and exchanging the confidence limits for risk difference.

^b^ NNT = 1 / (risk difference comparing with vs without text intervention). Probability(office visit | no intervention) = 1. Probability(office visit | intervention) = sensitivity × prevalence + (1 – specificity) (1 − prevalence) = 1 × 0.58 + 0.53 × 0.42 = 0.80 = 80%. Risk difference = 1 – [sensitivity × prevalence + (1 – specificity) (1 – prevalence)]. NNT = 1 / [1 – (sensitivity × prevalence) + (1 – specificity) (1 – prevalence)].

## Discussion

Leveraging digital technologies to monitor patient-reported outcomes in oncology is well established^3,4,5^ but, to our knowledge, has not yet been applied as a tool to reduce treatment-related time burden. We show in this cross-sectional study that a simple text message–based questionnaire can accurately identify patients who are not experiencing symptoms of ICI toxic effects (100% negative predictive value, 0% false-negative rate). This implies that such patients with normal laboratory parameters can likely bypass the usual pretreatment office visit and proceed directly to ICI infusion with minimal risk of missing an immune-related adverse event. Although the modest specificity of 47% implies that the tool erroneously detects ICI toxic effects in some patients, this approach still offers the potential to reduce up to 20% of office visits (ie, number needed to text, 5). One limitation of this study is that it was performed at a single academic health system primarily enrolling White patients, potentially limiting its generalizability. Further work is needed to integrate a text message–based triage system into routine care and understand its impact on the patient experience. A prospective randomized trial directly comparing this instrument vs the standard office visit with the outcomes of total care time, patient and clinician acceptability, and feasibility is under way.

## Notes

Supplement. **eAppendix.** Triage Instrument
