# Supplementary material for: Accuracy of a Text Intervention to Minimize the Burden of Cancer Care Among Patients Treated With Immune Checkpoint Inhibitors
Source: JAMA Netw Open. 2022 Aug 29;5(8):e2228452. doi: 10.1001/jamanetworkopen.2022.28452 (PMC9425147; doi:10.1001/jamanetworkopen.2022.28452)
Supplement: Supplement. — eAppendix. Triage Instrument [file jamanetwopen-e2228452-s001.pdf]

Supplemental Online Content

Bange EM, Coughlin K, Li W, et al. Accuracy of a text intervention to minimize the burden of cancer care among patients treated with immune checkpoint inhibitors. *JAMA Netw Open*. 2022;5(8):e2228452. doi:10.1001/jamanetworkopen.2022.28452

eAppendix. Triage Instrument

This supplemental material has been provided by the authors to give readers additional information about their work.

© 2022 Bange EM et al. *JAMA Network Open.*

eAppendix. Triage Instrument
TIME Study Feedback Survey

You are receiving this survey because over the past 4 months, you enrolled in a study with Penn Medicine where a text message-based survey was utilized to assess your symptoms, with the goal of testing out a new approach to care and to minimizing the time burden of cancer.

We are hoping to receive feedback on this text message survey. We appreciate your time.

Did you receive the text-based survey asking about symptoms from your cancer treatment, sent 4 days prior to your infusion appointment?	◯ Yes side effects and<br>◯ No<br>◯ Not Sure
Did you complete the text-based survey asking about symptoms from your cancer treatment, sent 4 days prior to your infusion appointment?	◯ Yes side effects and<br>◯ No<br>◯ Not Sure
If no, why not? (Select all that apply.)	☐ Survey was too long<br>☐ I was too busy<br>☐ I did not receive it<br>☐ I did not understand it<br>☐ Other
Other:	______________________________
Did the text-based survey result in additional fees on your phone bill?	◯ Yes<br>◯ No<br>◯ Not Sure
If yes, how much was the additional fee?	◯ Less than $1<br>◯ $1-3<br>◯ More than $3<br>◯ No fee
When is your preferred time of day to receive the text-based survey? (Select all that apply.)	☐ Morning (7-10AM)<br>☐ Midday (10AM-2PM)<br>☐ Afternoon (2-5PM)<br>☐ Evening (5-8PM)
What is your preferred platform to receive the text-based survey? (Select all that apply.)	☐ Via phone call<br>☐ Via text message<br>☐ As a URL to a web-based survey
Imagine you were able to reduce the time spent at a typical oncology visit without impacting the quality of your visit. What is the minimum amount of time reduced that would have a positive improvement on your day?	◯ 15 min<br>◯ 30 min<br>◯ 45 min<br>◯ 60 min<br>◯ More than 60 min

.© 2022 Bange EM et al. *JAMA Network Open.*

If you could reduce the above amount of time spent on an oncology visit, would that time ideally be removed from:	◯ Commute<br>◯ Waiting to see provider<br>◯ Provider visit<br>◯ Waiting for chemo<br>◯ Other

Other:

.© 2022 Bange EM et al. *JAMA Network Open.*

Thank you for your feedback.

Please see below additional questions to collect demographic information. This information is not connected to you and its collection will not impact your care. Rather, we will use this data to assess and analyze the instrument and any barriers to its use.

What is your age?	______________________
What is your current gender identity?	◯ Male<br>◯ Female<br>◯ Trans male<br>◯ Trans female<br>◯ Other
Other gender identity:	______________________
Which race do you identify with? (Select all that apply.)	☐ White<br>☐ Black or African American<br>☐ Asian<br>☐ Native Hawaiian or Pacific Islander<br>☐ American Indian or Alaskan Native<br>☐ Other
Other race:	______________________
Where is your primary residence?	◯ Philadelphia County<br>◯ Pennsylvania (not in Philadelphia County)<br>◯ New Jersey<br>◯ New York<br>◯ Delaware<br>◯ Maryland<br>◯ Other
Other location:	______________________
What is your annual individual income from all sources?	◯ $0-20,000<br>◯ $20,001-40,000<br>◯ $40,001-60,000<br>◯ $60,001-80,000<br>◯ $80,001-100,000<br>◯ $100,001+
What was your highest level of education completed?	◯ Less than 8th grade<br>◯ Some high school but did not receive diploma<br>◯ High school diploma/GED<br>◯ technical, or trade school training<br>◯ Some college but did not receive a diploma<br>◯ Associates Degree (AA or AS)<br>◯ Bachelor's degree (for example BA or BS)<br>◯ Some graduate (Masters or Doctorate)<br>◯ Masters Degree<br>◯ Professional Degree (for example MD, JD, DD, PharmD<br>◯ Doctorate degree (PhD, EdD)

.© 2022 Bange EM et al. *JAMA Network Open.*

What is your marital status?	◯ Single (living alone)<br>◯ Married (living together, cohabitating, common-law)<br>◯ Separated (legal or non-legal)<br>◯ Divorced<br>◯ Widowed

.© 2022 Bange EM et al. *JAMA Network Open.*

Supplement. Immunotherapy toxicity instrument sent to patients by text message.

1. In the last 7 days, have you had a fever of 100.4 F degrees or greater?

Possible Response: Y (1) / N (0)

2. In the last 7 days, how much did FATIGUE, TIREDNESS, OR LACK OF ENERGY INTERFERE with your usual or daily activities?

Possible Response: Not at all (0); A little bit (1); Somewhat (1); Quite a bit (1); Very much (1)

3. In the last 7 days, did you have ANY new or worsening PAIN (CHEST PAIN, ABDOMINAL PAIN, JOINT PAIN, MUSCLE PAIN, OR OTHER)?

Possible Response: Y (1) /N (0)

4. In the last 7 days, did you have any new or worsening SKIN CHANGES (RASH, BLISTERS, SKIN PEELING, OR OTHER)?

Possible Response: Y (1) /N (0)

5. In the last 7 days, what was the severity of your ITCHY SKIN at its WORST?

Possible Response: None (0); Mild (1); Moderate (1); Severe (1); Very severe (1)

6. In the last 7 days, have you had any new or worsening DIZZINESS OR LIGHT-HEADEDNESS?

Possible Response: Y (1) /N (0)

7. In the last 7 days, have you had any new or worsening NUMBNESS OR TINGLING IN YOUR HANDS OR FEET?

Possible Response: Y (1) /N (0)

8. In the last 7 days, have you had any new or worsening WEAKNESS OF YOUR ARMS OR LEGS?

Possible Response: Y (1) /N (0)

9. In the last 7 days, did you have any new or worsening BLURRY VISION?

Possible Response: Y (1) /N (0)

10. In the last 7 days, did you have any new or worsening HEADACHE?

.© 2022 Bange EM et al. *JAMA Network Open.*

Possible Response: Y (1) /N (0)

11. In the last 7 days, did you have any new or worsening SHORTNESS OF BREATH?

Possible Response: Y (1) /N (0)

12. In the last 7 days, have you had any new or worsening COUGH?

Possible Response: Y (1) /N (0)

13. In the last 7 days, how often did you have NAUSEA?

Possible Response: Never (0); Rarely(1); Occasionally(1); Frequently(1); Almost constantly(1)

14. In the last 7 days, how often did you have VOMITING?

Possible Response: Never (0); Rarely (1); Occasionally (1); Frequently (1); Almost constantly (1)

15. In the last 7 days, how OFTEN did you have LOOSE OR WATERY STOOLS (DIARRHEA)?

Possible Response: Never (0); 1-2 times per day (1); 3-4 times per day (1); 5-6 times per day (1); Almost constantly (1)

16. Do you have any other symptoms you wish to report?

Possible Response: Y (1) /N (0)

Please type your symptom below and rate the severity on a scale of: None, Mild, Moderate, Severe, Very Severe

.© 2022 Bange EM et al. *JAMA Network Open.*
